# Recommendation of the German Society of Hospital Hygiene (DGKH): Prevention of COVID-19 by virucidal gargling and virucidal nasal spray – updated version April 2022

**DOI:** 10.3205/dgkh000416

**Published:** 2022-07-07

**Authors:** Axel Kramer, Maren Eggers, Martin Exner, Nils-Olaf Hübner, Arne Simon, Eike Steinmann, Peter Walger, Paula Zwicker

**Affiliations:** 1 Institute of Hygiene and Environmental Medicine, University Medicine Greifswald, Greifswald, Germany; 2 German Society of Hospital Hygiene, Berlin, Germany; 3 Labor Prof. Gisela Enders MVZ GbR, Stuttgart, Germany; 4 Institute for Hygiene and Public Health, University Hospital Bonn, Bonn, Germany; 5 Central Unit for Infection Prevention and Control, University Medicine Greifswald, Greifswald, Germany; 6 Pediatric Oncology and Hematology, Children’s Hospital Medical Center, Saarland University Hospital, Homburg/Saar, Germany; 7 Institute of Hygiene and Microbiology, Department for Molecular & Medical Virology, Ruhr-University Bochum, Bochum, Germany; 8 Bonn, Germany; 9 Section Clinical Antisepsis of the German Society of Hospital Hygiene, Berlin, Germany

**Keywords:** guideline, virucidal gargling, virucidal nasal spray, SARS-CoV-2, COVID-19

## Abstract

The German Society of Hospital Hygiene develops guidelines, recommendations and standard operation procedures on a voluntary basis, published on the DGKH-website (https://www.krankenhaushygiene.de/).

The original German version of this recommendation was published in April 2022 and has now been made available to the international professional public in English. Evaluating the current data on the efficacy of virucidal gargle/mouthwash solutions and nasal sprays against SARS-CoV-2 *in vitro* and in clinical trials, conducted with preventive or therapeutic objectives, recommendations are given for the prevention of COVID-19. The following areas are considered:

- Protection of the community when regional clusters or high incidences of infection become known
- Protection of the community at low risk of infection
- Pre-exposure prophylaxis for the protection of healthcare workers
- Post-exposure prophylaxis

## Abstract (de)

Die Deutsche Gesellschaft für Krankenhaushygiene (DGKH) erarbeitet Leitlinien, Empfehlungen und Standardarbeitsanweisungen auf freiwilliger Basis, die auf der DGKH-Website veröffentlicht werden (https://www.krankenhaushygiene.de/).

Die deutsche Originalfassung dieser Empfehlung wurde im April 2022 veröffentlicht und wird jetzt auf Englisch der internationalen Fachöffentlichkeit zur Verfügung gestellt. In Auswertung der aktuellen Datenlage zur Wirksamkeit viruzider Gurgel-/Mundspüllösungen und Nasensprays gegen SARS-CoV-2 *in vitro* und in klinischen Studien, die mit präventiver oder therapeutischer Zielsetzung durchgeführt wurden, werden Empfehlungen zur Prävention von COVID-19 gegeben. Dabei werden folgende Bereiche berücksichtigt:

- Schutz der Bevölkerung bei Bekanntwerden regionaler Cluster oder hohem Infektionsgeschehen
- Schutz der Bevölkerung bei geringem Infektionsrisiko
- Präexpositionsprophylaxe zum Schutz des Personals im Gesundheitswesen
- Postexpositionsprophylaxe

## 1. Introduction

To prevent COVID-19, all available hygienic measures must be implemented to protect the community and, in particular, highly exposed individuals such as medical personnel. Personal behaviors, including adherence to distance rules and social contact restrictions, wearing of nose-mouth mask, vaccination, virucidal prevention in the nasopharynx, healthy diet, and physical activity, are primarily the responsibility of the individual, but are significantly influenced by intergenerational coexistence, living space, educational level, socioeconomic status, and, in the case of vaccination, additional misleading misinformation based on conspiracy theories [1], [2], [3], [4]. Particularly in the generation over 60 years old, government recommendations for infection-preventive behavior correlate significantly with their implementation in the everyday life [5]. Therefore, the prevention potential of virucidal antisepsis in the nasopharynx, which has received too little attention so far, should be given the importance it warrants in public relations and recommendations to popularize it as an easy-to-implement preventive measure. This can be used to supplement infection prevention for occupationally exposed personnel, but also for contact persons living in the same household with SARS CoV-2 infected persons or persons suffering from COVID-19. 

The following statements reflect the current state of knowledge regarding the efficacy of antiseptics in the prevention of SARS-CoV-2 infections.

The entry site for SARS-CoV-2 is the nasopharyngeal cavity. In the delta variant the viral load in nasopharyngeal swabs is higher than in saliva [6], in the omicron variant vice versa [7], so that both areas must be included in virucidal antisepsis. Presumably, infection can also occur via the eye, provided droplets reach the eye directly [6], [7]. Because a large proportion of infected individuals release the virus before the onset of initial symptoms, protective measures that reduce the viral load at the ports of entry, i.e., the nasopharynx, are useful because the likelihood of contracting the infection increases with the extent of exposure. Because the initial viral load also influences the severity of disease after infection, virucidal antisepsis at the ports of entry may even mitigate manifesting infection during the course of disease [8], [9], [10], [11]. 

Gargling was long used to reduce upper respiratory tract infections and to treat bacterial/viral infections (e.g., strep throat, common cold), but has now fallen out of fashion. Hand washing with soap and water and gargling with saline solution were recommended to the population in Germany by the State Health Council as a preventive measure as early as during the Spanish flu in 1918 [12]. In former East Germany, school children were advised to gargle with diluted potassium permanganate solution when entering summer holiday camp [13]. Furthermore, the beneficial soothing effect of salty air on the respiratory tract has been known for centuries. It stimulates the natural self-cleaning of the respiratory tract and prevents the mucous membranes from desiccating. In addition, moistening the mucous membranes of the mouth and nose prevents the adhesion of viruses and is therefore preventively effective even without the use of solutions/sprays with their own antiviral efficacy [14], [15]. In contrast to Europe, daily gargling with saline has a long tradition in Japan and in Korea for the prevention of respiratory infections. Gargling was increasingly promoted by the Japanese Ministry of Health, Labour and Welfare during the 2009 H1N1 swine flu pandemic and has been explicitly recommended as a daily routine for the general public since the COVID-19 pandemic began [13].

To exploit antiseptic use in the nasopharynx as an easily realizable measure and prophylactic instrument for the prevention and control of COVID-19 in Germany, knowledge on the virucidal efficacy and preventive use options of nasal sprays and gargle solutions is summarized below. A decisive advantage of antiseptic measures is that if a gargle solution or nasal spray is proven to be effective against SARS-CoV-2, the effect is directed against all variants of the virus and at the same time other respiratory enveloped viruses, such as influenza viruses.

## 2. State of knowledge on the virucidal efficacy of mouthrinse/gargle solutions and nasal sprays

To limit the bibliography, sources cited in the review by Kramer and Eggers [13] are not explicitly listed, only indirectly with reference to this review.

### 2.1 In vitro efficacy 

Efficacy against SARS-CoV-2 has been proven for the following agents or formulations [13], [16], [17], [18], [19], [20], [21], [22], [23], [24], [25], [26], [27], [28], [29], [30], [31], [32]:

- *PVP-iodine* ≥0.23% with an exposure time of 15–60 s, applicable in the oral cavity, nasal cavity, and eye.
- Mouth- or gargle rinses based on
  - *essential* *oils*,
  - *dequalinium* *chloride*,
  - without or with content of ethanol/*benzalkonium chloride* (Dequonal^®^)*, *
  - *phenoxyethanol + octenidine* (Octenisept*^®^*),
  - *ethanol + ethyl laurylarginate, *
  - *delmopinol hydrochloride, *
  - *di-potassium oxalate, *
  - *cetylpyridinium chloride* (CPC), and
  - PHTALOX^®^, a phthalocyanine derivative.

*PVP-iodine* from 0.5% induces complete virus inactivation within 15 s, which is achieved by 70% ethanol after 30 s [33].

For mouthrinses based on *essential oils*, complete inactivation of SARS-CoV-2 was verified both with alcohol content (Listerine^®^ Cool Mint) and without alcohol content (Listerine^®^ Cool Mint mild taste) [34]. In contrast, mouthrinses based on *hydrogen peroxide* (HPO), *polihexanide, chlorhexidine digluconate* (CHG), or *octenidine* (the latter without combination with phenoxyethanol) were not sufficiently effective [23], [33], [34]. Consistently, CHG was also ineffective in the oral cavity [35]. Studies in which CHG had been found to be effective [36] were apparently based on incomplete neutralization of CHG adsorbed to virus and only simulated efficacy. Results differ for *stabilized hypochlorite* [32].

*Green tea, pomegranate juice*, and *aronia juice* are effective against various pathogens of respiratory infections; however, the efficacy is lower than that of the mouthrinses mentioned above [13]. *Aronia juice* has now also been shown to be effective against SARS-CoV-2 [37]. *Green tea* reduces the titer of SARS-CoV-2 by 80% after 1 min [37]. For sage extract, efficacy against influenza and human corona viruses has been demonstrated, and sage extract was therapeutically as effective as the antiviral aciclovir against herpes labialis; thus, it is highly likely that it is also effective against SARS-CoV-2 [13].

Nasal sprays based on *saline* (0.9%), *xylometazoline hydrochloride* (0.1%), and the combinations *hydroxypropylmethylcellulose/succinic acid/disodium succinate* or *Galphimia glauca/ Luffa operculata/ Sabadilla* were ineffective. The combination of *sodium hypochlorite* (<0.08%) with *lithium magnesium sodium silicate* achieved a reduction factor of 2.2 [38], which, however, is not considered sufficient for virucidal activity. Since the product forms a gel matrix that may interfere with the virus, an effect may be achieved *in vivo* if need be. However, the tolerability of sodium hypochlorite for long-term use requires clarification.

### 2.2 Preventive efficacy in in vivo and in-use studies

#### Gargling

Gargling with *hypertonic saline* (2%–3%) 3 times/d significantly shortened the duration of illness from viral influenza, with the reduction in viral shedding reducing the incidence of illness by 35%, even among people living in the same household. Since saline is not virucidal *in vitro* [36], hypochlorite is probably formed intracellularly due to the increased chlorite availability; it is also possible that viral adherence is reduced.

*Green tea* also reduced the manifestation of viral influenza by 30% compared with water or no gargling (5 studies; [13]).

However, a higher protective effect is achieved by using virucidal antiseptics. For example, rinsing the oral cavity with 1% *PVP-iodine* solution for 1 min significantly reduced the amount of SARS-CoV-2 in saliva with high viral load for the duration of 3 h [39]. In another study, both PVP-iodine and CPC significantly decreased the recovery of viral load compared with rinsing with water for the duration of the 6 h-study [35].

*In vivo*, the lack of efficacy of 1% *HPO* [40] and the low efficacy of *CHG* [41], [42] were confirmed. In a RCT, after a single mouth rinsing, virus levels were insufficiently reduced, by only 61–89% after 15 min, regardless of whether 0.12% CHG, 1% *HPO*, *saline*, or 0.5% *PVP-iodine* [43] was used, i.e., a single rinse is not sufficient for a sustained effect. Similarly, a single rinse with *sorbitol* and *xylitol* (Linolasept^®^ mouthwash) reduced the viral load by 90%, that means 1 lg [44].

#### Nasal spray

Although *carragelose* does not inactivate SARS-CoV-2 *in vitro* [38], it inhibits viral replication *in vitro* [45], [46]. In 3 studies (n>600), application as nasal spray significantly reduced the duration of illness and the number of patients with symptoms in respiratory infections caused by human rhino, corona, and influenza A viruses, respectively. The viral load was significantly lower in the verum group than in the placebo group [47]. The hypothesized mode of action is that the high molecular weight polymer of the sugar-like molecule galactose forms a mucoadhesive layer on the nasal mucosa that interacts with the virus.

### 2.3 Therapeutic efficacy in in-use studies

#### Gargling

When the combination of ethanol with *essential oils* was used for mouthrinsing in cases of *H. simplex*, HSV-1 and HSV-2 were no longer detectable, in contrast to the control (water) [13]. This confirms the expectation that antiseptics effective *in vitro* against enveloped viruses are also effective preventively and possibly even therapeutically when used in humans.

In stage 1 patients (presymptomatic stage 1–2 d before first symptoms after infection of COVID-19), viral clearance was significantly increased by both 1% *PVP-iodine* and the combination of ethanol with *essential oils* compared with tap water on days 4, 6, and 12 [9]. Also, in a small case-study in Spain, 1% PVP-iodine decreased the viral load in COVID-19 patients [39]. In a RCT (n=303 for each group) including patients who had a positive PCR on the first symptom day of COVID-19, 1% *PVP-iodine* solution was applied as a gargling solution and as nasal and eye drops (control lukewarm water) every 4 h for 4 weeks immediately after confirmed diagnosis. In the treatment group, morbidity and mortality were significantly reduced on days 3, 5, and 7 [43]. Thyroid hormone levels were not affected.

In an intervention study in children age 10 years or older (n=995), with protective measures otherwise identical to those in the control group, 2-month application of *PHTALOX* 3–5 times/d for 1 min reduced the incidence of COVID-19 by 54% (p=0.076) [48].

In a RCT (n=88 each), gargling 3 times daily for 7 d with *ß-cyclodextrin* Citrox significantly reduced SARS-CoV-2 in saliva in asymptomatic and mild COVID-19 4 h after initial application [49].

#### Nasal spray

In colds, *carragelose* (Algovir^®^ cold spray: 1.2 mg carragelose +0.5% NaCl) significantly reduced both the number of people who fell ill and the duration of illness (3 studies; [13]).

### 2.4 Concluding evaluation of the study situation

*HPO* and *CHG* show little or no effectiveness *in vitro*, which was confirmed clinically. *CPC* (0.04–0.075%) and *essential oils* are moderately to highly effective *in vitro* and also clinically effective [32]. Considering the overall data, *PVP-iodine* based antiseptics (0.5–1%) are superior to the other agents listed. In the oral cavity, gargle solutions based on *essential oils* can be assumed to have a comparable effect.

Currently, further randomized trials with new active ingredients are being conducted to be able to implement the easily realizable prevention potential of virucidal nasopharyngeal antisepsis in an even more targeted manner.

## 3. Risk assessment for long-term use

The use of *carragelose* (red algae extract), *saline, green tea, aronia juice,* and *essential oils* is safe without and with the addition of ethanol.

1.25 % *PVP-iodine* is tolerated in the nasal cavity without subjectively disturbing sensations and does not cause inhibition of cilia activity [50]. While PVP-iodine 2.5% causes severe eye burning, the concentration of 1.25% PVP-iodine is tolerated without irritation or damage [51], does not penetrate the anterior chamber of the eye [52] and does not affect thyroid function [53], [54].

PVP-iodine’s absorption during gargling has not been investigated. Under the worst case assumption of 10% absorption, a single gargle with 1.25% PVP-iodine would result in the absorption of about 1000 µg of iodine, which is 5 times the daily dietary iodine intake recommended by the WHO. Iodine-induced hyperthyroidism or hypothyroidism have been described in the context of topical applications only with excess exposures many times higher than those possible with gargling. Single case reports are available for urinary bladder or peritoneal irrigation or for irrigation of extensive wounds [55], [56], [57], [58]. In a review, Frank et al. [59] concluded that the use of PVP-iodine in the oral cavity in concentrations of up to 2.5% is safe for up to 5 months.

Since March 2020, pre-exposure prophylaxis has been performed at Greifswald University Medicine with 1.25 % *PVP-iodine* solution and, in case of contraindication, with mouthrinse based on ethanol/essential oils. So far, there has been no sign of incompatibilities.

## 4. International recommendations and recommendations derived from the evaluation of the current state of knowledge for Germany

### 4.1 Protection of the population when regional clusters or high incidences of infection become known

#### Gargle

So far, recommendations have only been made for the pandemic situation or in hotspots. However, in view of the decline in the incidence of the disease, it seems reasonable to make recommendations for the endemic situation as well. 

Since no commercially available antiseptics with a reduced content of PVP-iodine are available in Germany, suggestions are made for self-preparation by diluting the commercially available oral antiseptic Betaisodona Oral Antiseptic (contains 7.5% v/v PVP-iodine and 36% v/v ethanol). Since the diluted solution is stable for only a short time, dilutions must always be prepared fresh.

- Preparation 0.23% solution:
  - Variant A: add 1 teaspoon of Betaisodona Oral Antiseptic to water glass half filled (100 ml) with lukewarm water.
  - Variant B: to prepare a smaller amount, e.g. to fill a spray applicator for application in the nose, 1 teaspoon of mouth antiseptic + 5 teaspoons of water).

- Preparation 1.25% solution for pre- or post-exposure prophylaxis:
  - Variant A: Pharmacy preparation according to New Prescriptions Formulary (NRF 15.13, [66]).
  - Variant B: Own preparation – add 3 teaspoons of Betaisodona Oral Antiseptic to a water glass half-filled (100 ml) with lukewarm water.

##### Japan

Gargle in the morning and evening with 0.23% *PVP-iodine* solution.

##### Germany

Gargle in the morning and evening with the combination of *essential oils* with ethanol (e.g., Listerine^®^ Cool mint). For people with alcohol intolerance or with mucosal sensitivity, the formulation without alcohol (Listerine^®^ Cool Mint mild flavor) should be used instead of the combination of *essential oils* with alcohol.

Otherwise healthy children are not at risk from acute SARS-CoV-2 infection (most have a mild disease course or are asymptomatic) [60]. Effective gargling is usually possible with some practice only once they have reached school age. Because of the better taste, *green tea* or *aronia juice* are more suitable for children.

#### Nasal cavity 

##### Japan

Morning and evening spraying with 0.23% *PVP-iodine* solution into both nostrils with simultaneous inhalation. 

##### Germany

In the absence of a *PVP-iodine* based nasal spray, use a *Carragelose*^®^-based nasal spray (e.g. Algovir^®^ cold spray) in the morning and evening; probably more effective is 0.23% PVP-iodine solution (self-production see above).

### 4.2 Protection of the community at low risk of infection

The following recommendations are derived for Germany.

#### Mouth rinse/gargle

*Rhythm:* Morning and evening, 3 times/d if possible, and additionally after eating meals together or other communal activities in elderly-care facilities or in rehabilitation facilities, at family gatherings (to the extent currently permitted), at professional group meetings, in schools and kindergartens, religious services and other religious occasions.

*Solutions:* *Saline solution* (preparation: Dissolve level teaspoon of saline in 100 ml of lukewarm water, put about amount of a shot glass in the mouth, interrupt gargling each time before inhaling, repeat process for about 3 min, finally spit out gargling solution). 

Alternatively, gargle with green tea, sage tea (preparation: pour about 3 g sage leaves with 150 ml boiling water, infuse for 10 min, pour tea through strainer, gargle with sage solution while still warm) or mouthwash based on essential oils.

#### Nasal cavity

*Rhythm:* Morning and evening 

*Agent:* Nasal spray based on *Carragelose*^®^ (e.g., Algovir^®^ cold spray), if possible, 3 times/d and in addition after eating meals together or other communal activities in elderly-care facilities or in rehabilitation facilities, at family gatherings (to the extent currently permitted), at professional group eetings, in schools and kindergartens, religious services and other religious occasions.

*Solutions: Saline solution *as an unpreserved product and without added decongestants (e.g., Hysan^®^ Salinspray^®^ or Rinupret^®^). 

Alternatively, prepare solution as for gargling (see above) and draw it into the nose by inhalation.

### 4.3 Pre-exposure prophylaxis for the protection of healthcare workers before aerosol producing interventions 

Before dental treatment, intubation, rhinoscopy, and bronchoscopy [64, 65], virucidal antisepsis in the oral cavity or vestibule nasi is recommended to reduce the viral load for the duration of the treatment. The patient is instructed to rinse the oral cavity thoroughly, spit out the solution, and then gargle. For nasal access, the use of 1.25% PVP-iodine solution as a spray is additionally recommended.

#### Belgium

Gargle with 1 % *PVP-iodine* [13].

#### Portugal, Malta

Gargle with 0.2 % *PVP-iodine* [13].

#### World Health Organization 

Gargle with 0.2 % *PVP-iodine* [61].

#### Germany

Gargling with 1.25% PVP-iodine solution, alternatively an essential oil based mouth rinse. 

In case of contraindications to iodine (hyperthyroidism, autonomous adenoma of the thyroid gland, iodine allergy), formulations based on* essential oils* can be considered. 

### 4.4 Postexposure prophylaxis

After unprotected known contact with SARS-CoV-2-positive for 14 d 1.25% *PVP-iodine* solution; if iodine is contraindicated, gargling with mouth wash based on essential oils with or without content of ethanol + nasal spray with 1.25% PVP-iodine solution (own production see above); *hypochlorite*-based nasal spray (e.g. Plasma Liquid nasal spray gel).

After eye contamination, rinse once with 1.25% PVP-Iodine solution. 

After accidental injury with risk of infection by SARS-C0V-2 PVP-I as alcoholic formulation (e.g. Braunoderm® or Betaseptic®).

## Notes

### Competing interests

The authors declare that they have no competing interests.

## Erratum

Affiliation update Peter Walger
